# Potential added value of combined DPYD/DPD genotyping and phenotyping to prevent severe toxicity in patients with a *DPYD* variant and decreased dihydropyrimidine dehydrogenase enzyme activity

**DOI:** 10.1177/10781552211049144

**Published:** 2021-11-19

**Authors:** Charlotte W Ockeloen, Aron Raaijmakers, Manon Hijmans-van der Vegt, Jörgen Bierau, Judith de Vos-Geelen, Annelieke ECAB Willemsen, Bianca JC van den Bosch, Marieke JH Coenen

**Affiliations:** 1 Department of Human Genetics, 6034 Radboud University Medical Center, The Netherlands; 2 Faculty of Science, 6029 Radboud University, The Netherlands; 3 Department of Medical Oncology, 6034 Radboud University Medical Center, The Netherlands; 4 Department of Clinical Genetics, Erasmus Medical Centre, Rotterdam, The Netherlands; 5 Department of Clinical Genetics, 199236 Maastricht University Medical Center+, The Netherlands; 6 Department of Internal Medicine, Division of Medical Oncology, GROW – School for Oncology and Developmental Biology, 199236 Maastricht University Medical Center+, The Netherlands; 7 Department of Internal Medicine, 3913 Tergooi MC, The Netherlands; 8 Department of Human Genetics, 6034 Radboud University Medical Center, Radboud Institute for Health Sciences, The Netherlands

**Keywords:** *DPYD*, DPD, DPD deficiency, decreased DPD activity, pharmacogenetics, fluoropyrimidines, fluoropyrimidine toxicity

## Abstract

Decreased dihydropyrimidine dehydrogenase enzyme activity is associated with severe fluoropyrimidine-associated toxicity. Four clinically relevant variants in the *DPYD* gene are associated with decreased dihydropyrimidine dehydrogenase activity. However, only ∼25% of *DPYD* variant carriers show a decreased dihydropyrimidine dehydrogenase activity in peripheral blood mononuclear cells.

**Objective:**

To investigate if dihydropyrimidine dehydrogenase phenotyping has added value when combined with *DPYD* genotyping in predicting fluoropyrimidine-related toxicity.

**Methods:**

Retrospective cohort study in which treatment and toxicity data were collected of 228 patients genotyped for four *DPYD* variants and phenotyped using an *ex vivo* peripheral blood mononuclear cell assay.

**Results:**

Severe toxicity occurred in 25% of patients with a variant and normal dihydropyrimidine dehydrogenase activity, in 21% of patients without a variant and with decreased dihydropyrimidine dehydrogenase activity, and in 29% of patients without a variant and with normal dihydropyrimidine dehydrogenase activity (controls). The majority of patients with a variant or a decreased dihydropyrimidine dehydrogenase activity received an initial dose reduction (68% and 53% vs 19% in controls) and had a lower mean dose intensity (75% and 81% vs 91% in controls). Fifty percent of patients with a variant and decreased enzyme activity experienced severe toxicity, despite the lowest initial dose and whole treatment dose intensity. They also experienced more grade 4/5 toxicities.

**Conclusions:**

Our results indicate that a combined genotype–phenotype approach could be useful to identify patients at increased risk for fluoropyrimidine-associated toxicity (e.g. patients with a variant and decreased dihydropyrimidine dehydrogenase activity). Because the group sizes are too small to demonstrate statistically significant differences, this warrants further research in a prospective study in a larger cohort.

## Introduction

Fluoropyrimidines (fluorouracil (5-FU) and its prodrug capecitabine) are commonly used in the treatment of several types of malignancies.^1–3^ Previous studies have shown that non-individualized fluoropyrimidine treatment causes severe toxicity in 6–34% of patients (grade ≥3, based on the Common Terminology Criteria for Adverse Effects (CTCAE) grading-system).^4–7^ Toxic effects of fluoropyrimidines mainly include stomatitis, mucositis, myelosuppression, and hand-foot syndrome. Many different factors including age, sex, renal function, liver function, co-medication, and other environmental factors are thought to influence the risk of toxicity.^8,9^

Over 80% of the active 5-fluorouracil (5-FU) is metabolized in the liver.^10^ The most important enzyme in the metabolism of fluoropyrimidines is dihydropyrimidine dehydrogenase (DPD).^11^ At least 3–5% of the Caucasian population has a decreased or absent DPD enzyme activity.^12,13^ As these findings strongly depend on methods used to measure DPD activity, considerably higher numbers of patients with decreased DPD activity have also been reported.^14^ Several studies have shown a link between a decreased DPD enzyme activity and severe fluoropyrimidine toxicity. Between 36 and 59% of the patients who experience severe fluoropyrimidine toxicity (grade ≥3) have a decreased DPD enzyme activity.^15–17^ Only in a minority of individuals, the reduced DPD activity can be explained by genetic variants in the *DPYD* gene.^14,18,19^

The European Medicines Agency recommends *DPYD* genotyping prior to fluoropyrimidine treatment after publication of a prospective trial, demonstrating that *DPYD* genotype-based dose reductions improved patient safety during fluoropyrimidine treatment.^20^ The four clinically relevant genetic variants in the *DPYD* gene that are known to be associated with a decreased DPD activity are DPYD*2A, DPYD*13, c. 2846A>T, and c.1129-5923C>G.^18,19,21^ The allele frequency of these variants is relatively low in the Caucasian population (between 0.03% and 1.35%).^22^ The gene activity score (GAS) is often used to translate the *DPYD* genotype into a DPD phenotype to describe DPD activity and for dose recommendations.^23^ This translation of *DPYD* genotypes to their functional activity is based on *in vitro* and *in vivo* studies and the subsequent GAS ranges from 0 (no DPD activity) to 2 (normal DPD activity). For heterozygous carriers of any of the clinically relevant variants, the Dutch Pharmacogenetics Working Group (DPWG) guidelines now recommend a dose reduction of 50%,^24,25^ instead of 50% for variants with a GAS of 1.0 and 25% for a GAS of 1.5 as was recommended before.^23^ This change in dose recommendation is supported by the Clinical Pharmacogenetics Implementation Consortium (CPIC) in the most recent update of their guideline for fluoropyrimidines and *DPYD*.^26^ Other rare and common *DPYD* genetic variants have been reported but are not included in the current international dosing guidelines due to insufficient evidence for functionality.^18,27^ When it is not possible to calculate the GAS based on *DPYD* genotype, for example when two variants might be located on the same allele, the DPWG recommends to determine DPD activity and adjust the initial dose based on available data.

*DPYD* genotyping is widely used globally. However, DPD enzyme activity has been underexplored. We previously showed that only 25% of patients with a decreased DPD enzyme activity carries one of the four *DPYD* variants.^18^ So although *DPYD* genotyping (of four variants) has proven to be useful in preventing toxicity and is cost-effective,^28,29^ the majority of the patients with a reduced enzyme activity might remain unidentified and are possibly susceptible to severe fluoropyrimidine toxicity.

So far, one trial describing a combined genotype–phenotype approach has been published.^14^ In this prospective trial, the occurrence of severe toxicity (early grade 4–5 toxic events) was compared between two groups. The first group was screened for DPD deficiency using a multi-parametric approach: determining uracil and dihydrouracil concentrations in blood, along with demographic characteristics, and *DPYD* genotyping. In the second group no pre-treatment testing was performed. The authors conclude that multiparametric pre-therapeutic DPD deficiency screening significantly avoided early severe life threatening toxic events. To our knowledge, no publications exist investigating the added value of the combined genotype–phenotype approach compared to *DPYD* genotyping alone, which is already widely implemented. Therefore, the aim of our retrospective study was to describe the value of combined *DPYD* genotyping and DPD phenotyping in predicting fluoropyrimidine-related toxicity in an existing hospital population, reflecting clinical practice.

## Methods

### Study design and group characteristics

This retrospective cohort study was performed in a single academic hospital in the Netherlands. It was approved by the local medical ethical committee. All 228 participants in this study were adults (≥18 years) diagnosed with cancer that were treated with fluoropyrimidines, either with capecitabine or 5-FU intravenously. The patients in this study were of different ethnic backgrounds, although the majority was Caucasian. Details on ethnicities will not be discussed because this was poorly reported. Fluoropyrimidine treatment was only given when the treating physician assessed the patient's condition to be sufficient, based on clinical presentation and laboratory parameters. Previous systemic therapy was allowed with resolved adverse events to grade ≤1. The patients received treatment in the period from January 2014 to December 2019. In case patients received more than one fluoropyrimidine treatment line in this period, all treatments were included in the analysis. This patient cohort is partly overlapping with the cohort described in a previous study which investigated the relationship between genetic variants in *DPYD* and DPD enzyme activity.^18^ All patients in this study were genotyped for the four clinically relevant *DPYD* variants and phenotyped with a DPD enzyme-activity assay using *ex vivo* peripheral blood mononuclear cells (PBMCs). The present study subsequently analysed treatment and toxicity data of these (and additional) patients. Patients with incomplete results of genotyping and phenotyping were excluded.

Dose adjustments were made based on genotype and phenotype results by the treating physicians according to national guidelines.^25^ In our analysis, the dose recommendations from the DPWG guidelines valid at the time of treatment were used. In some patients, due to clinical urgency, treatment had to be started right away without the genotyping and phenotyping results available; these patients were given a “safe start” (treatment with a lower dose, awaiting results of genotyping and phenotyping).

### DPYD gene variant and DPD enzyme activity assays

Both the *DPYD* gene analysis and enzyme activity test were performed in one academic hospital. DNA was isolated according to standard methods and DNA of the patients was analyzed using Sanger sequencing for the four clinically relevant *DPYD* variants: DPYD*2A (NM_000110.4(DPYD):c.1905+1G>A), DPYD*13 (NM_000110.4(DPYD):c.1679T>G), c. 2846A>T (NM_000110.4), and c.1129-5923C>G (NM_000110.4). An extensive description of the genotyping methods was previously published.^18^ In our study *1/*1 refers to patients who are not carriers of any of the four tested variants.

The DPD enzyme-activity assay using *ex vivo* PBMCs was performed with isolated lymphocytes. Patients were classified as having a decreased DPD enzyme activity when the enzyme activity was below 8.69 nmol/mg protein/h (<70% of the mean DPD activity in our reference cohort, which is 12.4 nmol/mg protein/h). The reference cohort and phenotyping methods are described in a previous publication.^18^

### Toxicity grading

The toxicity was graded on a 1–5 scale according to the CTCAE criteria, version 5.0.^4^ All adverse events that were possibly, probably, or definitely related to the fluoropyrimidine treatment, as described by the physician or qualified nurse practitioner, were registered. Severe toxicity refers to CTCAE grade 3 or higher. All ≥ grade 3 events were included, clinical as well as laboratory abnormalities. Radiotherapy was mentioned as concomitant treatment when this was applied during fluoropyrimidine treatment or up to two weeks before treatment.

### Patient groups

Based on the presence of *DPYD* variants and DPD enzyme activity measured in PBMCs, the patient cohort was divided into four groups. The first group was the control group, consisting of patients who carry none of the four tested *DPYD* variants and in whom a normal DPD activity was measured in PBMCs (*DPYD*^variant_no^*-*DPD^normal_activity^). The patients of the second group carried one of the four clinically relevant *DPYD* variants and had a normal DPD activity in PBMCs (*DPYD*^variant_yes^**-**DPD^normal_activity^). The third group consisted of patients who did not carry one of the four *DPYD* variants, but did have a decreased DPD activity in PBMCs (*DPYD*^variant_no^**-**DPD^low_activity^). The fourth group contained the patients who carried one of the four *DPYD* variants, as well as a decreased DPD activity in PBMCs (*DPYD*^variant_yes^*-*DPD^low_activity^).

The total group of patients carrying a variant (regardless of DPD activity) was designated *DPYD*^variant_yes^, the total group of patients with decreased DPD activity (with or without a *DPYD* variant) was designated DPD^low_activity^.

## Results

### Relevant cohort characteristics

A total of 228 patients treated with fluoropyrimidines were included in this study, consisting of 182 patients in the *DPYD*^variant_no^-DPD^normal_activity^ group, 16 in the *DPYD*^variant_yes^**-**DPD^normal_activity^ group, 24 in the *DPYD*^variant_no^-DPD^low_activity^ group, and 6 in the *DPYD*^variant_yes^*-*DPD^low_activity^ group. We included all patients with a *DPYD* variant and/or decreased DPD activity, and the majority of *DPYD*^variant_no^*-*DPD^normal_activity^ patients who were randomly selected. No significant difference in demographics, tumor type, number of treatment cycles, or occurrence of radiotherapy treatment was found between the groups (Table 1).

**Table 1.** Patient characteristics.

	*DPYD*^variant_no^ DPD^normal_activity^ (*n* = 182)	*DPYD*^variant_yes^ DPD^normal_activity^ (*n* = 16)	*DPYD*^variant_no^ DPD^low_activity^ (*n* = 24)	*DPYD*^variant_yes^ DPD^low_activity^ (*n* = 6)	*p*-value	Total (*n* = 228)
Male gender	106 (58.2%)	9 (56.3%)	14 (58.3%)	2 (33.3%)	0.72	131 (57.5%)
Age	62.5 (10.8)	66.3 (6.3)	62.0 (9.83)	59.2 (10.5)	0.44	62.6 (10.4)
BSA	1.91 (0.21)	1.92 (0.21)	1.94 (0.20)	1.94 (0.12)	0.91	1.91 (0.21)
Tumor type					0.91	
• Colorectal cancer	120 (65.9%)	11 (68.8%)	16 (66.7%)	4 (66.7%)		151 (66.2%)
• Pancreatic cancer	16 (8.8%)	1 (6.3%)	4 (16.7%)	0 (0%)		21 (9.2%)
• Metastatic breast cancer	16 (8.8%)	2 (12.5%)	1 (4.2%)	1 (16.7%)		20 (8.8%)
• Other	30 (16.5%)	2 (12.5%)	3 (12.5%)	1 (16.7%)		36 (15.8%)
Treatment^a^					0.47	
• Capecitabine • 5-FU	150 (82.4%)	12 (75%)	18 (75%)	6 (100%)		186 (81.6%)
	32 (17.6%)	4 (25%)	6 (25%)	0 (0%)		42 (18.4%)
Starting dose					<0.001	
• 100% of standard dose	148 (81.3%)	6 (37.5%)	13 (54.2%)	1 (16.7%)		168 (73.7%)
• 75% of standard dose	27 (14.8%)	8 (50.0%)	8 (33.3%)	2 (33.3%)		45 (19.8%)
• 50% of standard dose	2 (1.1%)	1 (6.3%)	3 (12.5%)	2 (33.3%)		8 (3.5%)
• Other	5 (2.7%)	1 (6.3%)	0 (0%)	1 (16.7%)		7 (3.1%)
Radiotherapy	34 (18.7%)	3 (18.8%)	1 (4.2%)	1 (16.7%)	0.30	39 (17.1%)
Number of treatment cycles^a^	5.4 (3.7)	5.13 (2.68)	5.67 (2.76)	4.17 (2.56)	0.034	5.4 (3.25)
*DPYD* variant					<0.001	
• No-variant • DPYD*2A het. • c.2846A>T het. • DPYD*13 het. • c.1129-5923C>G het.	182 (100%)	0 (0%)	24 (100%)	0 (0%)		206 (90.4%)
	0 (0%)	1 (6.3%)	0 (0%)	2 (33.3%)		3 (1.3%)
	0 (0%)	5 (3.3%)	0 (0%)	2 (33.3%)		7 (3.1%)
	0 (0%)	1 (6.3%)	0 (0%)	1 (16.7%)		2 (0.9%)
	0 (0%)	9 (56.3%)	0 (0%)	1 (16.7%)		10 (4.4%)
DPD enzyme activity (nmol/mg protein/h)	15.96 (4.43)	14.08 (4.85)	6.69 (1.25)	6.02 (2.08)	<0.001	14.59 (5.3)
• Normal activity	182 (100%)	16 (100%)	0 (0%)	0 (0%)	<0.001	198 (87%)
• Decreased activity^b^	0 (0%)	0 (0%)	24 (100%)	6 (100%)		30 (13%)

BSA: body surface area; 5-FU: 5-fluorouracil; het.: heterozygote; DPD: dihydropyrimidine dehydrogenase; *DPYD*: gene encoding DPD protein.

Relevant characteristics with corresponding percentages of the total number of patients are shown for the categorical data. The mean and standard deviation are shown for the numerical data since these data follow a normal distribution (age, BSA, and number of treatment cycles, DPD enzyme activity). Groups: DPD^normal_activity^ = normal DPD enzyme activity, DPD^low_activity^ = decreased DPD enzyme activity below 8.69 nmol/mg protein/hour, *DPYD*^variant_no^ = no variant, *DPYD*^variant_yes^ = carrier of one of four variants affecting DPD activity (DPYD*2A (c. 1905+1 G>A; rs3918290), c. 2846A>T p.(Asp949Val); rs67376798), DPYD*13 (c. 1679T>G p.[Ile560Ser]; rs55886062), and c.1129-5923C>G (rs75017182)).

a This applies only to the first treatment plan, any other treatment plans received by patients were not included.

b A patient was considered to have a decreased DPD activity when the DPD enzyme activity was below 8.69 nmol/hour per mg protein.

Colorectal cancer was the most frequent cancer reported in 66% of the patients, followed by pancreatic and breast cancer. The vast majority of the patients received treatment with capecitabine (82%), while a smaller proportion was treated with intravenous 5-FU. Standard initial treatment dose was received by 74% of the patients, while 26% of the patients received an initial dose reduction, median 75% (range 25–84%). The average body surface area (BSA) was 1.9 m^2^, which is slightly higher than that of the average Dutch adult population (1.7 m^2^).^30^

### DPYD variant carriers and decreased DPD enzyme activity measured in PBMCs

**Figure 1.**
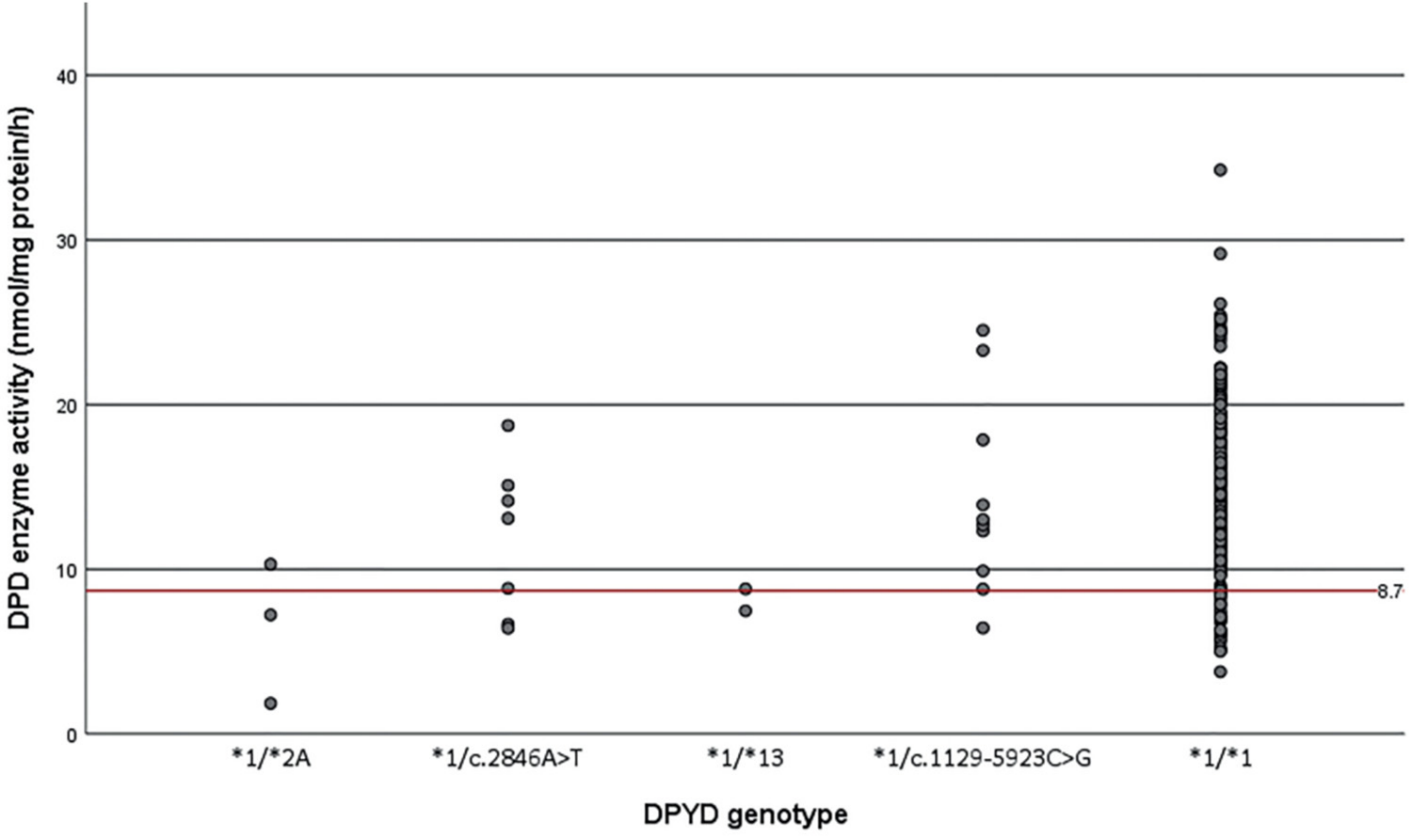
Results of the DPD enzyme activity assay using ex vivo PBMCs are shown on the *y*-axis, the different *DPYD* genotypes that are known to influence DPD enzyme activity can be seen on the *x*-axis: DPYD*2A/*1 (c. 1905+1 G>A; rs3918290), c. 2846A>T/*1 p.(Asp949Val); rs67376798), DPYD*13/*1 (c. 1679T>G p.[Ile560Ser]; rs55886062), c.1129-5923C>G/*1 (rs75017182) and *1/*1. The dots represent patients who are all heterozygous carriers of one of the four variants. The red line represents the cut-off value for decreased DPD activity (<8.69 nmol/mg protein/h).

DPD: dihydropyrimidine dehydrogenase; PBMC: peripheral blood mononuclear cell.

Twenty-two patients carried one of the four *DPYD* gene variants associated with decreased DPD activity and 32 patients had a decreased DPD activity measured in PBMCs. No homozygous *DPYD* variant carriers were identified in our cohort, nor patients with zero DPD enzyme activity. 6/22 heterozygous *DPYD* variant carriers (27%) showed a decreased DPD enzyme activity measured in PBMCs (DPD activity was below 8.69 nmol/per mg protein/hour) (Figure 1). More specifically, 29% of the c.2846A>T variant carriers and 10% of the c.1129-5923C>G variant carriers showed a decreased DPD enzyme activity. Of the DPYD*2A and DPYD*13 variant carriers, 67% and 50% of patients had a decreased DPD enzyme activity, respectively. There was a broad range of DPD activity in non-variant carriers.

### Dose reductions and toxicity in different patient groups

Dose reductions and the occurrence of severe toxicity in the different patient groups are shown in Table 2. The different types of toxicity and toxicity scores are shown in Supplemental Table 2.

**Table 2.** Dosage and occurrence of severe toxicity of the four different patient groups.

	*DPYD*^variant_no^ DPD^normal_activity^	*DPYD*^variant_yes^ DPD^normal_activity^	*DPYD*^variant_no^ DPD^low_activity^	*DPYD*^variant_yes^ DPD^low_activity^	*DPYD* ^variant_yes^ total	DPD ^low_activity^ total	All patients
Number of patients	182	16	24	6	22	30	228
Mean dose intensity of all treatments	91%	78%	84%	67%	75%	81%	80%
Dose reduction due to adverse effects	61 (34%)	3 (19%)	9 (38%)	2 (33%)	5 (23%)	11(37%)	75 (33%)
Complete stop of fluoropyrimidine treatment**^a^** due to adverse effects	41 (23%)	5 (31%)	6 (25%)	3 (50%)	8 (36%)	9 (30%)	55 (24%)
Dose reduction and/or dose discontinuation due to adverse effects	76 (42%)	8 (50%)	7 (29%)	1 (17%)	9 (41%)	8 (27%)	92 (40%)
Initial dose reduction (first cycle of first treatment)	34 (19%)	10 (63%)	11 (46%)	5 (83%)	15 (68%)	16 (53%)	60 (26%)
Overall grade ≥3 toxicity	52 (29%)	4 (25%)	5 (21%)	3 (50%)	7 (32%)	8 (27%)	63 (28%)
Overall grade ≥3 toxicity for patients with an initial dose reduction**^b^**	7 (21%)	2 (20%)	3 (27%)	2 (40%)	4 (27%)	5 (31%)	14 (23%)
Overall grade ≥3 toxicity for patients with a standard initial dose**^c^**	45 (30%)	2 (33%)	2 (15%)	1 (100%)	3 (43%)	3 (21%)	50 (30%)

DPD: dihydropyrimidine dehydrogenase; *DPYD*: gene encoding DPD protein.

The dose intensity is defined by the percentage of the standard dose for the specific treatment that was given. The percentages between brackets apply to the total number of patients per group except for ^a^. Groups: DPD^normal_activity^ = normal DPD enzyme activity, DPD^low_activity^ = decreased DPD enzyme activity below 8.69 nmol/mg protein/hour, *DPYD*^variant_no^ = no variant, *DPYD*^variant_yes^ = carrier of one of four variants affecting DPD activity (DPYD*2A (c. 1905+1 G>A; rs3918290), c. 2846A>T p.(Asp949Val); rs67376798), DPYD*13 (c. 1679T>G p.[Ile560Ser]; rs55886062), and c.1129-5923C>G (rs75017182)).

^a^ Complete stop of fluoropyrimidines during whole treatment.

^b^ The percentages between brackets apply to the group total of “initial dose reduction.”

^c^ The percentages between brackets apply to the group total of “standard initial dose.”

The mean dose intensity was highest in the *DPYD*^variant_no^*-*DPD^normal_activity^ group (91%), followed by 84% in the *DPYD*^variant_no^*-*DPD^low_activity^, 78% in the *DPYD*^variant_yes^**-**DPD^normal_activity^, and 67% in the *DPYD*^variant_yes^**-**DPD^low_activity^ group. The majority of the *DPYD*^variant_yes^ and DPD^low_activity^ patients received an initial dose reduction (63% and 53% vs 19% of the patients without a variant and with normal DPD activity).

Overall, the percentage of patients who developed severe toxicity was 32% in *DPYD* variant carriers (*DPYD*^variant_yes^), 27% in patients with decreased DPD activity (DPD^low_activity^), and 29% in the *DPYD*^variant_no^*-*DPD^normal_activity^ group. More specifically, ≥grade 3 toxicity occurred in 25% of the *DPYD*^variant_yes^*-*DPD^normal_activity^ group and in 21% of the *DPYD*^variant_no^**-**DPD^low_activity^ group. In the *DPYD*^variant_yes^**-**DPD^low_activity^, severe toxicity occurred in 3/6 (50%) of patients. The latter are discussed in more detail below. Table 3 gives an overview of the mean dose intensities and frequencies of ≥grade 3 toxicity in the four patient groups.

**Table 3.** Schematic overview of the mean dose intensity and frequency of ≥grade 3 toxicity in the four different patient groups.

	Normal DPD activity	Low DPD activity	
*DPYD* variant	*N* = 16	*N* = 6	*N* = 22
	Mean dose intensity 78%	Mean dose intensity 67%	
	Severe toxicity 25%	Severe toxicity 50%	
*DPYD* non-variant	*N* = 182	*N* = 24	*N* = 206
	Mean dose intensity 91%	Mean dose intensity 84%	
	Severe toxicity 29%	Severe toxicity 21%	
	*N* = 198	*N* = 30	*N* = 228
Total

In the *DPYD*^variant_no^*-*DPD^normal_activity^ group, 21% of patients with initial dose reduction experienced severe toxicity versus 30% treated with a standard initial dose. About half of the *DPYD*^variant_no^*-*DPD^normal_activity^ patients who received an initial dose reduction (18/34) had a “safe start” (a lower dose awaiting genotyping and phenotyping results), the remaining for other reasons, for example general condition of the patient or laboratory abnormalities (Supplemental Table 1).

In the *DPYD*^variant_yes^*-*DPD^normal_activity^ group, results were similar: 20% of patients with an initial dose reduction experienced severe toxicity, which was 33% in patients treated with a standard initial dose. In the *DPYD*^variant_no^**-**DPD^low_activity^ group, however, 27% of patients treated with a lower initial dose experienced severe toxicity, versus 15% of patients who were treated with a standard initial dose.

### Toxicity in DPYD^variant_yes^-DPD^low_activity^ group

Fifty percent of the patients with a clinically relevant variant and a decreased enzyme activity (*DPYD*^variant_yes^*-*DPD^low_activity^ group) experienced severe toxicity; even though they received the lowest initial dose and whole treatment dose intensity. Furthermore, 50% (3/6) of the patients in this group completely discontinued fluoropyrimidine treatment due to adverse events. This is much less frequent in the other groups, in which 23–31% of patients discontinued treatment due to adverse events (Table 2). Of the patients with a variant and decreased DPD activity, 40% (2/6) with an initial dose reduction experienced ≥grade 3 toxicity, as did the one patient who received a standard initial dose.

Of the three patients with severe toxicity in the *DPYD*^variant_yes^*-*DPD^low_activity^ group, two experienced grade 4 toxicity and one patient died (grade 5 toxicity). In the other groups, grade 4 and 5 toxicity was very rare (Supplemental Table 2). Of note, two of these three patients from the *DPYD*^variant_yes^**-**DPD^low_activity^ group who experienced severe toxicity, initially received a higher dose than recommended at that time by the DPWG guidelines (Supplemental Figure 1). The patients who have a GAS of 1.0 were recommended to receive an upfront dose reduction of 50%; however, two of the three patients with a GAS of 1.0 received 75% and 100% of the standard dose, respectively. In both patients, this was because test results were not available prior to treatment (because treatment had to be started immediately). The patient who received the initial dose of 75% (awaiting results of genotyping and phenotyping) died. When the patient was identified as a variant carrier with reduced DPD enzyme activity, capecitabine was stopped completely. Shortly thereafter the patient was admitted because of deteriorating condition, neutropenic, and thrombocytopenic. The patient died of sepsis. This was likely secondary to intestinal mucositis, but the cause of the fulminant infection could not be identified with autopsy. The patient who received the standard initial dose (100%) was tested for DPD deficiency after development of serious toxicity and capecitabine was discontinued.

## Discussion

There are very few studies investigating toxicity in patients who are genotyped and phenotyped for DPD prior to fluoropyrimidine treatment. The results of this retrospective cohort study suggest that patients carrying a *DPYD* variant who also have a decreased DPD enzyme activity, might have a high risk of toxicity even with a reduced fluoropyrimidine dose intensity. These patients also seem to have more grade 4–5 toxicity. Our results indicate that in patients with a known *DPYD* variant, the risk of severe toxicity could be even higher when the DPD enzyme activity is also low.

Our results indicate that DPD phenotyping is beneficial in identifying *DPYD* variant carriers with a decreased DPD enzyme activity. Formal conclusions with regards to the added value of pre-treatment phenotyping in patients without a *DPYD* variant in individualizing fluoropyrimidine treatment require a larger sample size. Currently, a prospective clinical trial is ongoing in the Netherlands to investigate this (clinicaltrials.gov NCT04194957). In addition, information about cost-effectiveness of additional DPD phenotying is lacking. Nevertheless, there are important advantages of phenotyping, one being able to identify patients at risk of severe toxicity who cannot be identified with standard *DPYD* genotyping (which was 9% of patients in a previous study^18^). These include patients with rare *DPYD* variants or patients of other ethnic backgrounds. Phenotyping is also useful when a patient carries two *DPYD* variants associated with decreased DPD activity; in this case phenotyping is recommended by the DPWG.^25^

Some drawbacks exist with regards to the utility of the DPD phenotyping method in the clinic.^19^ The *ex vivo* PBMC assay is considered a labor-intensive method for which one needs special equipment, which is not available in every hospital, which makes it difficult to implement this method on a large scale. A cut-off value of <70% below the population mean is used to classify patients as having a decreased DPD activity,^15^ which predicts fluoropyrimidine-associated toxicity,^16,17^ but no prospective study has been performed to determine whether this is the optimal threshold. For patients with a decreased DPD activity but no *DPYD* variant, there is little evidence with regards to optimal dosing strategies. The CPIC guidelines recommend a dose reduction in patients with a DPD enzyme activity <70% of normal DPD activity in the population, but no clear cut-off values are available to determine the optimal starting dose.^26^ In one review several phenotype-based approaches are compared.^19^ Only for DPD enzyme measurement in PBMCs a dosing strategy is established. This method calculates the starting dose in patients with decreased DPD activity using the following formula; the percentage remaining DPD activity compared to normal/average multiplied by the standard dose (% activity present × normal dose). This method, however, has not yet been validated. In summary, the clinical validity of PBMC DPD activity has been well established, but there is no clear consensus on the optimal dose adjustment strategy based on the enzyme activity.

The four clinically relevant *DPYD* gene variants correlate differently with phenotypic DPD enzyme activity. Although the groups are small, we found that carriers of the c.2846A>T or c.1129-5923C>G variant rarely have a decreased DPD activity measured in PBMCs (29% and 10%), while carriers of the DPYD*2A or DPYD*13 variant more frequently have a DPD activity below the threshold (67% and 50%). This is in agreement with a previous study, in which it is shown that DPYD*2A and DPYD*13 carriers have a significantly lower Dihydrouracil/Uracil ratio indicative of DPD deficiency.^23^

In the total *DPYD*^variant_yes^ group, 32% (7/22) experienced severe toxicity. Only 27% of the patients who are carriers of a clinically relevant *DPYD* variant have a decreased DPD activity measured in PBMCs. In a larger study, also including the patients from this study, we observed the same.^18^ For the patients with a variant and a normal DPD activity it might be possible to treat with a higher dose as the activity is in the normal range despite the variant. This is something that warrants additional research.

In *DPYD*^variant_yes^ and DPD^low_activity^ patients, the initial dose was reduced more frequently and the whole treatment dose intensity was lower, compared to the *DPYD*^variant_no^-DPD^normal_activity^ patients. Despite this, the differences in overall grade ≥3 toxicity seem to be small between these groups. This could be caused by the reduced dose; if *DPYD*^variant_yes^ and DPD^low_activity^ patients would be treated with standard doses, more toxicity would be expected. No large difference in treatment alterations due to adverse effects in patients with and without initial dose reduction seem to be present, but the groups are too small to demonstrate significance (Supplemental Table 3).

Patients in the *DPYD*^variant_no^-DPD^low_activity^ group who started treatment with the standard initial dose unexpectedly experienced less severe toxicity than *DPYD*^variant_no^*-*DPD^low_activity^ patients with a standard initial dose. This could be due to chance as a result of the small group sizes or perhaps an indication that the threshold for low DPD activity is not optimal. The difference in severe toxicity between patients with or without initial dose reduction in the different groups cannot be explained by concomitant treatment with radio-chemotherapy (known to result in decreased risk of toxicity). Moreover, the difference cannot be explained by other factors such as whole treatment dose intensity, type of medication used, or DPD activity relative to the cut-off value (Supplemental Table 4).

Our study has several limitations. First of all, the number of patients is too small for statistical analyses. This is an observational study, and our results warrant further research in a larger, prospective study. There might be a selection bias because not all *DPYD*^variant_no^*-*DPD^normal_activity^ patients were included; however, the included patients were randomly selected and there is no significant difference in group characteristics. Another limitation of this study is that patients with a variant or decreased DPD activity did not always receive the recommended dose reduction according to existing guidelines, because sometimes *DPYD/*DPD testing was performed after start of treatment. Ideally, treatment should be started after obtaining genotyping and phenotyping results, but this is not always possible due to the urgency of chemotherapeutic treatment in case of severe illness. In these cases, dose adjustments are made in the second cycle based on test results.

Nevertheless, other aspects of our study are very important. This study was performed using an existing hospital population, which reflects common practice (in one hospital). Finally, this is one of the few study populations combining genotype and phenotype data, as well as treatment and toxicity data.

In most hospitals, *DPYD* genotyping prior to fluoropyrimidine treatment is standard practice nowadays. DPD enzyme activity measurement is used less frequently in the Netherlands. Using a combined genotype–phenotype approach could be useful in identifying patients with a *DPYD* variant that have an even higher risk of severe fluoropyrimidine-induced toxicity. Furthermore, phenotyping can identify patients who do not carry one of the four *DPYD* variants but do have a decreased, or no DPD activity (which also includes patients who carry a rare *DPYD* variant not included in standard genotyping). Further studies in larger cohorts are needed to provide statistical substantiation and to investigate which starting dose alterations should be considered in the *DPYD* variant patients with decreased DPD enzyme activity. Finally, our results underscore the importance of obtaining genotyping and phenotyping results *before* start of treatment, although this might not be possible in all cases.

## Supplemental Material

sj-docx-1-opp-10.1177_10781552211049144 - Supplemental material for Potential added value of combined DPYD/DPD genotyping and phenotyping to prevent severe toxicity in patients with a *DPYD* variant and decreased dihydropyrimidine dehydrogenase enzyme activity Click here for additional data file. Supplemental material, sj-docx-1-opp-10.1177_10781552211049144 for Potential added value of combined DPYD/DPD genotyping and phenotyping to prevent severe toxicity in patients with a *DPYD* variant and decreased dihydropyrimidine dehydrogenase enzyme activity by Charlotte W Ockeloen, Aron Raaijmakers, Manon Hijmans-van der Vegt, Jörgen Bierau, Judith de Vos-Geelen, Annelieke ECAB Willemsen, Bianca JC van den Bosch and Marieke JH Coenen in Journal of Oncology Pharmacy Practice
