# Supplementary material for: Potential added value of combined DPYD/DPD genotyping and phenotyping to prevent severe toxicity in patients with a DPYD variant and decreased dihydropyrimidine dehydrogenase enzyme activity
Source: J Oncol Pharm Pract. 2021 Nov 19;29(1):5–13. doi: 10.1177/10781552211049144 (PMC9749565; doi:10.1177/10781552211049144)
Supplement: sj-docx-1-opp-10.1177_10781552211049144 - Supplemental material for Potential added value of combined DPYD/DPD genotyping and phenotyping to prevent severe toxicity in patients with a DPYD variant and decreased dihydropyrimidine dehydrogenase enzyme activity [file sj-docx-1-opp-10.1177_10781552211049144.docx]

**Supplementary Material**


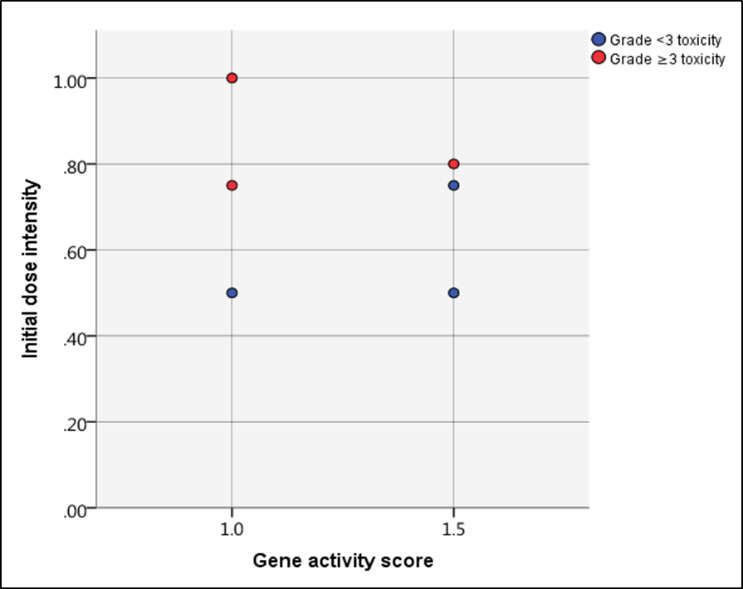


Dose

recommendation DPWG

**Supplementary Figure 1.** Gene activity scores and toxicity scores of *DPYD*^variant_yes^DPD^low_activity^ patients.

The gene activity score (GAS) of patients with one of the four *DPYD* variants and a decreased DPD enzyme activity (*DPYD*^variant_yes^DPD^low_activity^) is compared to their initial dose intensity (first cycle of the first treatment). The Dutch Pharmacogenetics Working Group dose recommendations for the different *DPYD* variants are also shown. *DPYD*^variant_yes^DPD^low_activity^ patients who started with an initial dose intensity higher than recommended by the DPWG experienced more grade ≥3 toxicity than patients who started on or below the recommended dose.

|  | ***DPYD^variant_no^***  **DPD*^normal_activity^*** | ***DPYD^variant_yes^***  **DPD*^normal_activity^*** | ***DPYD^variant_no^***  **DPD*^low_activity^*** | ***DPYD^variant_yes^***  **DPD*^low_activity^*** | **All patients** |
| --- | --- | --- | --- | --- | --- |
| **Number of patients** | 182 | 16 | 24 | 6 | **228** |
| **Initial dose reduction (first cycle of first treatment)** | 34 (19%) | 10 (63%) | 11 (46%) | 5 (83%) | 60 (26%) |
| **Dose reduction based on ^a^** |  |  |  |  |  |
| Safe start ^b^ | 18 (53%) | 2 (20%) | 3 (27%) | 2 (40%) | 25 (42%) |
| DPD activity | - | - | 6 (55%) | 2 (40%) | 8 (13%) |
| *DPYD* variant | - | 7 (70%) | - | - | 7 (12%) |
| Other | 16 (47%) | 1 (10%) | 2 (18%) | 1 (20%) | 20 (33%) |

**Supplementary Table 1.** Dose reduction specifics in the different patient groups.

The dose intensity is defined by the percentage of the standard dose for the specific treatment that was given. The percentages between brackets apply to the total number of patients per group except for ^a^. Groups: DPD^normal^ = normal enzyme activity, DPD^low^ = DPD enzyme deficiency, *DPYD*^normal^ = no variant, *DPYD*^variant^ = carrier of one of four variants affecting DPD activity (*DPYD**2A (c. 1905+1 G>A; rs3918290), c. 2846A>T p.(Asp949Val); rs67376798), *DPYD**13 (c. 1679T>G p.[Ile560Ser]; rs55886062) and c.1129-5923C>G (rs75017182)).

^a^ The percentages between brackets apply to the group total of ‘Initial dose reduction’.

^b^ Patients that received a ‘safe start’ already started treatment with a lower dose while waiting for the genotype and phenotype test results.

*Abbreviations*: DPD: dihydropyrimidine dehydrogenase; *DPYD*: gene encoding DPD protein.

| **Patient group** | **DPYD^variant_no^**  **DPD^normal_activity^** | **DPYD^variant_yes^**  **DPD^normal_activity^** | **DPYD^variant_no^**  **DPD^low_activity^** | **DPYD^variant_yes^**  **DPD^low_activity^** | **All patients** |  |
| --- | --- | --- | --- | --- | --- | --- |
| **Number of patients** | 182 | 16 | 24 | 6 | 228 |  |
| **Dose intensity first cycle of first treatment** | 95% | 82% | 86% | 72% | 83% |  |
| **Mean dose intensity of all treatments** | 91% | 78% | 84% | 67% | 80% |  |
| **Overall grade ≥3 toxicity** | 52 (29%) | 4 (25%) | 5 (21%) | 3 (50%) | 63 (28%) |  |
| **Overall grade ≥4 toxicity** | 7 (4%) | 0 (0%) | 1 (4%) | 2 (33%) | 10 (4%) |  |
| **Grade 5 toxicity** | 1 (0.5%) | 0 (0%) | 0 (0%) | 1 (17%) | 2 (0,9%) | |
| **Grade ≥ 3 gastrointestinal toxicity^a^** | 8 (4%) | 0 (0%) | 2 (8%) | 1 (17%) | 11 (5%) |  |
| **Grade ≥ 3 HFS^a^** | 5 (3%) | 0 (0%) | 2 (8%) | 0 (0%) | 7 (3%) |  |
| **Grade ≥ 3 hematological toxicity^a^** | 21 (12%) | 3 (19%) | 1 (4%) | 1 (17%) | 26 (11%) |  |
| **Anemia^b^** | 1 | 0 | 0 | 0 | 1 |  |
| **Neutropenia^b^** | 19 | 2 | 1 | 1 | 23 |  |
| **Thrombopenia^b^** | 2 | 1 | 0 | 0 | 3 |  |
| **Grade ≥ 3 other^a^** | 23 (13%) | 1 (6%) | 1 (4%) | 2 (33%) | 27 (12%) |  |

**Supplementary Table 2.** Toxicity scores in the different patient groups.

The dose intensity is defined by the percentage of the standard dose for the specific treatment that was given. The percentages between brackets apply to the total number of patients per group. Groups: DPD^normal^ = normal enzyme activity, DPD^low^ = DPD enzyme deficiency, *DPYD*^normal^ = no variant, *DPYD*^variant^ = carrier of one of four variants affecting DPD activity (*DPYD**2A (c. 1905+1 G>A; rs3918290), c. 2846A>T p.(Asp949Val); rs67376798), *DPYD**13 (c. 1679T>G p.[Ile560Ser]; rs55886062) and c.1129-5923C>G (rs75017182)). The toxicity was graded using the CTCAE.

^a^ The percentages between brackets apply to the total number of patients in that group.

*Abbreviations*: DPD: dihydropyrimidine dehydrogenase; *DPYD*: gene encoding DPD protein. HFS = hand-foot syndrome.

|  | ***DPYD*^variant_yes^ or DPD^low_activity^ patients**  **No treatment alterations due to adverse events** | ***DPYD*^variant_yes^ or DPD^low_activity^ patients**  **Dose reduction, discontinuation or complete stop due to adverse events** | **Total** |
| --- | --- | --- | --- |
| Initial dose reduction | 10 | 10 | 20 |
| No initial dose reduction | 12 | 7 | 19 |
| Total | 22 | 17 | 39 |

**Supplementary Table 3.**

Effects of initial dose reduction in patients with a *DPYD* variant or decreased DPD activity on treatment alternations due to adverse events. Initial dose reduction = reduction in the first cycle of first treatment, based on results of geno- and phenotyping, safe start, or other reasons, reflecting clinical practice.

|  | *DPYD*^variant_no^-DPD^low_activity^  (n = 24) | |
| --- | --- | --- |
|  | Standard initial dose | Initial dose reduction |
| Number of patients | 13 | 11 |
| Radio-chemotherapy | 0 (0%) | 1 (9%) |
| Mean dose intensity of all treatments | 94% | 72% |
| Medication  5-FU  Capecitabine | 4 (31%)  9 (69%) | 2 (18%)  9 (82%) |
| Mean DPD enzyme activity (nmol/mg protein/h)  Mean difference with deficiency cut-off value | 6.83  1.86 | 6.52  2.17 |

**Supplementary Table 4**. Initial dose characteristics *DPYD*^variant_no^-DPD^low_activity^, patients without a variant but with decreased DPD enzyme activity.

The dose intensity is defined by the percentage of the standard dose for the specific treatment that was given. The percentages between brackets apply to the total number of patients per group. Patient group: *DPYD(0)* = none of the four variants (*DPYD**2A (c. 1905+1 G>A; rs3918290), c. 2846A>T p.(Asp949Val); rs67376798), *DPYD**13 (c. 1679T>G p.[Ile560Ser]; rs55886062) and c.1129-5923C>G (rs75017182)), *DPD(-)* = reduced enzyme activity as measured in PBMCs, with a DPD-activity below the cut-off value of 8.69 nmol/hour per mg protein.

*Abbreviations*: DPD: dihydropyrimidine dehydrogenase; *DPYD*: gene encoding DPD protein.
